# Lecture on Phthisis Pulmonalis—Meningitis—Paralysis—Inflammation of the Heart and Its Membranes—Autopsy of Phthisis Complicated with Pleurisy, Pericarditis, and Endocarditis

**Published:** 1839-12-28

**Authors:** W. W. Gerhard


### CLINICAL LECTURE.

### PHILADELPHIA HOSPITAL.

*Wednesday, December* 18, 1839.

LECTURE ON PHTHISIS PULMONALIS—MENINGITIS—PARALYSIS—INFLAMMATION OF THE HEART AND ITS MEMBRANES—AUTOPSY OF PHTHISIS COMPLICATED WITH PLEURISY, PERICARDITIS, AND ENDOCARDITIS.

By W. W. Gerhard, M. D.

*No. 6—Winter Course.*

I will to-day conclude the subject of phthisis for the present, by showing you one or two cases of the disease, and saying a few words in relation to its treatment. I shall occupy the remainder of the hour with some cases of cerebral and cardiac disease, and the results of the post mortem examination of an individual who recently died of phthisis complicated with serous inflammations of the heart and lungs.

I will first present to you a case of phthisis, commencing in a different mode from any of which I have yet spoken. The patient was attacked about two years since with *coxalgia*, of which he has never entirely recovered. The treatment consisted in the use of blisters and a seton: the discharge from the latter, after having continued for some time, was allowed to cease. About eight weeks after, he was seized with cough, which still continues, with other signs of phthisis; for a few days of the period which has elapsed since the commencement of the cough, there has also been hæmoptysis. The phthisis in this case evidently commenced with a scrofulous disease of the hip-joint; for in two months after the discharge established for the cure of the coxalgia had ceased, the symptoms of phthisis began, and have since progressed in the irregular order. The scrofulous diathesis, therefore, before affecting the lungs, developed itself *externally*. Cases of this sort are by no means rare; the external scrofulous disease may be seated in other parts than the hip; sometimes, for example, it occus in the form of *fistula in ano*. An important practical question occurs in relation to such cases: Ought we to endeavour to cure the external disease? If we do, there is great danger that the irritation may be transferred to the lungs, and lead to the development of tubercles: on the contrary, if we suffer the disease to proceed unmolested, the constitutional irritation arising from it may destroy the patient, or give rise indirectly to the formation of tubercles in the lungs, by producing a condition of the system favourable to this result. The proper course would seem to be, not to check the external disease too suddenly, but, if possible, to subdue it by degrees. Some time ago we had a case here, which illustrated the effects of an opposite plan of treatment. The patient was first attacked with tubercular meningitis; after recovering from that, he had fistula in ano; this was cured, phthisis consequently supervened, and the man died. These cases are extremely common, and you will see many such in your practice. The case now before us is also one in point. The arrest of these external discharges may likewise give rise to other diseases, among which are diseases of the heart, and inflammation of the lungs; the latter in such cases being often of a more chronic character than in ordinary pneumonia.

*Case* 2.—We have here an example of phthisis occurring in old age; the patient is sixty-two years old. He has been employed in one of the oyster cellars of this city,—a situation, from its dampness, and also its darkness, extremely favourable to the development of phthisis. He has had cough for seven years, but it has never been severe till the commencement of the past summer. There is now well marked cavernous respiration, with pectoriloquy, and other signs of cavity in the upper lobes of both lungs. Phthisis occurring at such an advanced age is extremely rare. But experience shows us that no age is exempt from this disease. Tubercles are found even in the fœtus, and at every period of life. They are most frequently met with, however, about the fifth year, and afterwards from the fifteenth to the twenty-fifth. Cases of phthisis, or other tuberculous diseases, occurring in old persons, are much more frequently observed in hospitals than in private practice.

I next introduce these men, formerly patients, but now employed in the house, in whom cavities in the lungs have been more or less perfectly healed. The first is a case of phthisis, in which a cavity became cicatrized after it had continued to a very advanced stage, but was reproduced upon a second attack of the disease. The patient entered the hospital about three years since, with all the signs of a cavity in the right lung: dulness on percussion, cavernous respiration, &c., were very well marked. He remained in the wards for several months, during which time his condition was constantly improving: he was then discharged, and was, to all appearances, nearly well,—a small cavity, however, still remained, with slight cough and expectoration. After he had been out of the hospital about five months, he had an attack of intermittent fever, which continued for some weeks. After he recovered from the fever, he had a second attack of phthisis, or, as it were, a new crop of tubercles, for which he was under treatment in our wards. He is again much improved, though still feeble. The local signs indicate a partial consolidation of the lung by the process of cicatrization; thus, there is dulness on percussion, and a diminution of the natural vesicular respiration. These indications of a cicatrix exist where formerly were heard a strong cavernous respiration, and all the other signs of a cavity of considerable size. The cure in this case, then, is only partial.

But in the case which I now present to you, there has been a complete restoration. The patient, in the year 1835, had an attack of gangrene of the lungs, which continued for several months, with very fœtid expectoration, and all the other symptoms of this affection. The local signs indicated a cavity large enough to contain the fist. After a time the expectoration became muco-purulent,—a change which indicated an arrest of the gangrene, and the formation of a false membrane on the surface of the cavity. The man, as you see, is now stout, free from dyspnœa, and in every respect perfectly healthy. There has existed no scrofulous vice in the constitution, tending to reproduce the cavity, as happened in the preceding case. The disease was caused by cold and intemperance. As the man has now been well for four years, the cure may be considered complete.

You see, therefore, that it is possible to cure a cavity in the lungs, however infrequent may be the occurrence of such a result, especially in phthisis, where new crops of tubercles are so liable to form. The treatment, in all such cases, is entirely negative; there is, in fact, *no remedy* for the lesion. All that we can do is to palliate the symptoms, and support the constitution of the patient till nature, if she be so disposed, accomplishes the cure. Generally, the prognosis after a cavity has been once formed, is altogether unfavourable; we always look for the death of the patient, whether the cavity be the result of a tubercular deposition, or of any other lesion. But before the cavity is formed, our chances of success are much greater. In hospitals, however, our prospects, in either case, are far less encouraging than in private practice. We are entirely unable to adopt those measures which are most essential to a successful issue; we can only employ palliative remedies; in relation to food, clothing, air, exercise, &c., the means at our command are necessarily very limited and imperfect. In private practice, on the contrary, we are enabled more successfully to combat the general disease, by changing as far as possible the whole constitution of our patients; for this purpose, we direct a change of scenes and of climate by travelling, which is our principal reliance in such cases.

In the treatment of phthisis, you will find that there is great practical importance in the classification of the disease into several varieties, which I have called your attention to in preceding lectures. The inflammatory variety may frequently be arrested in the earlier stages by the ordinary antiphlogistic means which we employ in cases of simple inflammation. In that variety which commences slowly and gradually, on the contrary, we derive little or no aid from this plan of treatment. The treatment of phthisis, therefore, must be as various as the different forms of the disease. In the ordinary, slow cases, we must attempt to change, as it were, the whole being and nature of our patient, in the same manner as we do with regard to the mental constitution in the treatment of insanity. By thus producing a change in the constitution, we endeavour to cause the expulsion of the tubercular vice. I am not able to enter fully into this subject at present; nor, in the remarks which I shall hereafter make upon the treatment of phthisis, shall I attempt to give you more than the leading points,—to lay the foundation for a successful study of the subject. For the details, I must refer you to the different treatises which have been written on the disease.

At the last lecture, you will recollect, I introduced some cases of meningitis; the more severe of these cases I show you again to-day. When the patient was before you last week he was very ill, and had all the characteristic symptoms of meningitis; there was a distortion of the mouth, rigidity of the left arm, contraction of the pupil, delirium. These symptoms have since declined, and are now entirely gone. The contraction or distortion of the mouth is in such cases a valuable sign, and frequently gives us the key to the nature of the disease at the first sight of the patient: it is equally valuable, as you will see presently, in hemiplegia. The patient now suffers no pain, except in the region of the liver, and the adjacent portions of the abdomen,—the case being one of meningitis, co-existing with peritonitis. The tongue is of the natural appearance, and moist; skin moist and pleasant. The only symptom of the disease which still exists is an unnatural condition of the pulse: it is still rather slow and intermittent; it beats sixty-six in the minute; each pulsation is quick, but there is a long interval between them. The pulse, however, is by no means so slow as it is in the third stage of the disease, where there is coma. The treatment has consisted in a free bleeding from the arm at the commencement of the attack, followed by cups and blisters to the temples, cold applications, and the administration of one grain of calomel every hour. The mouth was slightly affected by the calomel, and the symptoms at once began to decline. General bleeding should be practised only in the early stages of the disease; afterwards cups and leeches should be employed, and repeated until the patient is weakened by the loss of blood, or the disease begins to abate. Cold applications to the head must be continued throughout the disease; bladders filled with powdered ice, or very cold water, may be employed, or vinegar and water, or evaporating lotions, such as diluted spirits. As regards mercurials in acute meningitis, I generally begin with a purgative dose of calomel, and afterwards give a grain every hour or two until slight ptyalism is produced. In chronic, or rather in mild cases, I give it in small doses. Blisters are particularly useful in this form of the affection; bleeding, of course, is not demanded to such an extent as in the acute form. Meningitis may generally be easily cured in its earlier stages; but after stupor has supervened, it becomes much more difficult, because an effusion of lymph has now taken place, and there is danger that the life may not be sustained until the effused matter is absorbed: hence the result is almost always fatal. In pleurisy, on the other hand, notwithstanding such effusions, a sufficient degree of motion to sustain respiration is retained, and the prospects of a favourable termination are, therefore, almost certain.

There is a variety of subacute meningitis, or simple congestion of the brain, (as it sometimes is,) occurring in persons accustomed to continued and intense mental application, as students, physicians, lawyers, and divines, in which improper treatment is sometimes productive of great mischief. In these cases, free bleeding is useless and injudicious; it weakens the patient, and renders him anemic, while it acts very slightly on the membranes of the brain. It is, therefore, only a palliative measure, which will prepare the patient for after treatment; the congestion still continues to occur, and on very slight causes. The treatment ought to be of a simple character, but continued for a considerable time to produce a permanent benefit. I generally direct the use of the warm bath two or three times a week; cold affusions to the head several times daily, to be continued for weeks, or months, if necessary; cups or leeches should be applied a few times at the beginning,—the former are generally preferable, and are vastly more within your reach. I also use gentle purgatives, as a means of producing revulsion; rhubarb will answer this purpose very well, either singly, or combined with aloes, or mercurials, or magnesia. Blisters are also very useful, especially when there is a tendency to epilepsy; they should be small, and repeatedly applied, either to the temples, or behind the mastoid process, according to the position of the pain: the latter is the best place for their application when the disease principally affects the base of the brain.

I next bring to your notice a case of inflammatory cardiac disease. The patient, a mulatto, aged twenty-two, enjoyed good health until about five months since. He can assign no cause for his present illness, which commenced about the month of July. He was seized, while in bed, with hæmoptysis and palpitation of the heart; the former was at first considerable, but, after some time, ceased; the palpitations have continued. No pain was complained of until after his entrance into the hospital; it is now constant, but is most severe during the day; he lies on the back with the head raised, and is unable to remain without great distress, in any other position. Some time ago his abdomen began to swell; afterwards his face and legs; there is slight cough and mucous expectoration. In November he went to the Dispensary in Fifth street, where he was bled, cupped, and blistered over the præcordial region, and got some pills of calomel and digitalis. This treatment produced considerable improvement in the symptoms. Afterwards he applied to a quack in the city, who placed him upon some stimulating treatment, under which he grew worse. He entered the hospital December 4th. Since that time the symptoms have continued pretty uniform: they consist of the primary local signs, and the general or secondary symptoms, produced by the obstruction of the circulation, which is the consequence of the disordered action of the heart. Attending first to the local signs, you will remark, even at a little distance from the patient, a considerable fulness or prominence in the præcordial region, extending over a much larger space than that naturally occupied by the heart. There is flatness on percussion throughout this region, the lungs being entirely pushed aside by the heart. There is a bellows or rasping sound in the first sound of the heart; the second is inaudible. The action of the heart was at first violent; it is now indistinct, and concealed by fluid in the pericardium: still we can perceive that it is irregular. The preceding signs indicate effusion into the pericardium, and thickening of the heart, with dilatation, arising from inflammation of this organ and its membranes. The bellows and rasping sounds are produced—1, by the spasmodic action of the heart; 2, by an obstruction to the passage of the blood, which is produced by thickening of the valves. As the valves are generally irregularly thickened, the current of blood is broken, and gives rise to a rough sound very like that produced by the passage of water through an irregular or broken pipe. The quick spasmodic contractions of the heart cause a considerable variety in the rapidity of passage and force of the blood, and therefore the sound changes frequently,—passing, at times, from the bellows sound to the rougher tone to which you give the name of rasping sound, and again subsiding to a common bellows murmur when the temporary increase in the rapidity of the heart's action has ceased.

The rough sound occurs during the systole of the heart, because it is directly dependent upon the ventricular contraction; but it may be produced either at the mitral or at the semilunar valves: when it occurs at the semilunar valve, you will almost always find that the second sound is lost or greatly impaired, because its production depends upon the integrity of this valve. When it is caused by regurgitation through the mitral valve, from deficiency in its surface, and consequent inaptness to close completely the orifice during the contraction of the heart, you may still distinguish the valvular arterial sounds. In this patient the second sound is so deficient that I infer that the semilunar valve is thickened and stiffened, as well as the mitral more or less altered.

The patient, you see, is young and stout, as are many patients affected with hypertrophy and dilatation following acute inflammation of the heart. These affections frequently arise from rheumatism, as is often the case with the inflammations of serous membranes generally; but, in the present instance, no such origin can be traced. Strange as it may seem, few patients die of the acute symptoms of cardiac diseases; it is the chronic forms of these affections that prove so distressing and fatal. Hence it is our duty always to arrest them, if possible, in the acute stage; when the disease has become chronic, we may be certain that an organic lesion of the heart has taken place, which is altogether incurable. In the treatment of this case, therefore, which was decidedly inflammatory, we have adopted the most active antiphlogistic measures from the commencement. The patient was first bled to the amount of 16 oz., and the bleeding has been several times repeated; cups have been twice applied, followed by blisters to the præcordial region; warm pediluvia have been employed, and the lowest diet only allowed. The following prescription was likewise continued until the mouth was slightly affected:

℞. Pulv. Digitalis gr. j.
Hydrarg. Submuriat., et
Pulv. Opii $\overline{aa}$ gr. $\frac{1}{3}$. M.
S. To be taken three times a day.

The patient's condition is now, in every respect, much improved; but, as there is certainly an organic change in the structure of the heart, we cannot hope for his entire recovery.

The last case which I bring before you is one of paralysis, probably caused by the pressure of a tumour on the right side of the brain, near the orbit. The patient is an old woman, who was admitted into the hospital a few days since; but no satisfactory history of her affection can be obtained from her. I can, therefore, only direct your attention to her actual state. The first symptom which I noticed when I first saw the patient was a slight distortion of the mouth, which at once led me to suspect the nature of the case. The mouth, you will perceive, is slightly drawn to the right side; the complete use of the right arm is retained, while the left is kept crossed on the chest, and is raised with much difficulty; the left leg also partakes of this rigidity and loss of power; the tongue is protruded imperfectly, and is now inclined a little to the left side,—two days since, it was to the other side. The direction of the tongue in cases of paralysis depends upon the particular muscle which may happen at the moment to be acting upon it; thus, when the digastricus of the healthy side acts, it will make the point of the tongue incline to the diseased side. When the patient speaks, you detect at the end of each word a peculiar thick, husky tone, which is so striking, that it may be properly called the *paralytic tone.* In addition to these symptoms, you perceive, at the slightest glance, that the right eye is very prominent, while the left retains its natural level: it is from this projection of the eye, especially, that I suspect the existence of a tumour pressing on the right hemisphere of the brain, and causing paralysis of the left side of the body; for paralysis and stiffness may both arise from simple softening of the brain. Indeed, in cases of tumours, the rigidity arises rather from the softening and inflammation around the tumour, than from the mere pressure of the mass.

In the few minutes which are left us, I will show you the lungs and heart of a subject who recently died in the wards of phthisis pulmonalis, complicated with pleurisy and inflammation of the membranes of the heart. You saw the patient last week, at which time I detailed to you the symptoms of the case, and the particular lesions which they indicated. Among other things, I called your attention to the pulse, which, notwithstanding the evident existence of pleurisy, was by no means of the strong, hard, full character, which it usually offers in this inflammation; it partook, in a great measure, of the nature of the irritable pulse of tubercular disease. It is by this character of the pulse that we are often enabled to determine at once whether pleurisy exists by itself, as a simple inflammation, or is attended with the deposition of tubercles, either in the lungs or the serous membranes. Pleurisy, however, may result in tuberculous disease, without being attended by the peculiar pulse of which I have spoken; but, in this case, the tubercles are not developed until after the pleurisy has disappeared.

The quick, irritated, and frequent pulse, together with the great heat of skin and profuse sweats, are the best, indeed almost the only means of distinguishing between simple pleurisy and that which is connected with a deposit of tuberculous matter. By attending to this circumstance you will rarely be mistaken; and I lately had the pleasure of assuring a personal friend whom I was called to visit at Washington, that a severe attack of pleurisy under which he laboured, was not connected with tubercles. If phthisis should follow such cases, it is usually a consequence of the pleurisy, perhaps, as some suppose, of the absorption of the purulent liquid; hence, even in the non-tuberculous variety of pleurisy, you should watch the termination of the disease.

The *left lung* is adherent throughout a considerable extent of its surface; some of these adhesions are evidently old, while others are very recent. The pulmonary pleura is very thick, opaque, and almost as dense as cartilage; it possesses this character over the whole lung, except that surface which faces the diaphragm. On cutting into the lung, several tubercles are seen immediately below the pleura; and near the summit there is a mass of tubercle occupying the whole of one of the lobules. Around this mass are seen numerous gray granulations, (the commencing stage of tubercle;) the lung is likewise congested, and infiltrated with bloody serum. *Right lung.*—About the middle of the upper lobe granulations are seen, but by no means so numerous as in the other lung. The rest of the lung is infiltrated with blood, and is of a dark shining colour at the summit, almost of the consistence of cartilage,—more so, indeed, than it is in pneumonia. This, likewise, is the commencing stage of a tubercular infiltration; but the infiltration occupies a different portion of the texture of the lung from that in which the granulations are found: the latter are seated in the vesicles,—the former pervades the cellular substance around them. This infiltration is the commencing stage of tubercle; and although not sufficiently advanced to be recognised by the physical signs, the peculiar characters of the pleurisy pointed out its true nature during life.

*Heart.*—The pericardium, before it was opened, contained two pints of slightly turbid serum; the membrane was highly injected, and of a bright red colour, which is now much less manifest; its transparency is much impaired, and its surface is roughened by the deposition of small masses of lymph. It was the presence of these little points of lymph which produced the creaking sound of the heart, of which I spoke at the last lecture. This ceased in a few days when the effusion increased. The lymph acts in the same way as a grain of sand in a piece of accurately adjusted machinery; it requires only a very small obstacle to the motion to produce a considerable grating or creaking whenever there is much friction. So these little fibrinous points, by opposing the partial revolution of the heart on its axis, gave rise to the creaking which was heard principally during the diastole, and at the commencement of the systole. You must have remarked that there is an immense disproportion between the amount of serum and of lymph which was effused in the course of the pericarditis. This is accounted for by the anemic condition of the patient's system; if his blood had contained more solid matter, we would doubtless have found more lymph on the surface of the pericardium, and less serum in its cavity.

A rasping or bellows sound of the heart was likewise heard during the man's illness. To account for this, we must examine the internal membrane of the heart. On opening the left ventricle, I find a yellowish coagulum of considerable firmness, such as is often found in endocarditis. The membrane lining the ventricle is opaque and slightly thickened. On opening the left auricle I see an immense vegetation, or cauliflower-like excrescence, occupying one portion of the mitral valve, with a partial destruction of the substance of the valve: this excrescence is much larger than any that I have ever before witnessed. It is about one-fourth of an inch thick; its edges very irregular and everted, forming the borders of an ulcer through which a finger can readily be passed from the ventricle to the auricle. The edges are everted towards the auricle, so that the total breadth of the excrescence is not less than an inch, and, of course, is nearly twice the breadth of the opening. The substance composing the edges of the ulcer is evidently the muscular substance of the heart, which is situated just beyond the fibrous portion of the valve; in fact, a portion of one of the columnæ carneæ projects inwardly towards the middle of the ulceration, and is divided directly across. The opposite lip of the valve is somewhat thickened and opaque, but free from vegetations. The internal membrane of the auricle is also much thickened, and of a dull, opaque, white colour.

The semilunar valves of the aorta are thickened at their base to a sufficient degree to afford some obstacle to the passage of the blood. The thickness of the left ventricle is half an inch at the middle; its cavity is about one-third, or a little more, greater than natural.

The cavity of the right ventricle presents an opaque whiteness of its lining membrane, which is extremely thickened in the half which is nearest to the tricuspid valve. The valve itself is thickened and opaque, but still flexible.

The impediment to the passage of the blood through the semilunar valve of the aorta may undoubtedly have caused some slight roughening of the first sound of the heart, but the chief cause of the morbid systolic sound was certainly the regurgitation from the left ventricle to the auricle. The size of the ulcerated opening was such that a very considerable current of blood passed in this manner, and was an evident cause of the morbid sound, which necessarily took place during the contraction of the ventricle, and the passage of the blood into the auricle through the morbid opening. During life this might have been distinguished with great precision, for the second sound of the heart was preserved until the last, and was very slightly altered. This was explained by the very slight alteration of the semilunar valves. The seat of the rasping sound corresponded to the middle of the heart, that is, to the part near the mitral valve, and from thence gradually diminished in ascending towards the aorta, or descending towards the apex of the heart. The character of the morbid sound varied from a rasping to a rough bellows sound, according to the degree of activity of the circulation, and the force of the ventricular contraction.

In a case like this, which is almost necessarily fatal, the precise situation of the most important lesion, would have been but of little practical utility; this, however, is far from being always the case. When we desire the greatest attainable accuracy in the diagnosis of valvular disease, we cannot do better than adopt the method suggested by my colleague, Dr. Pennock, who is extremely familiar with the pathology of cardiac disease. It consists in using a stethoscope composed of a flexible tube, about two feet long, with a hollow cone of an inch in diameter at the end, and the usual ear piece at the other, both formed of block tin; that is, the ordinary ear-trumpet, a little modified. The flexible tube neutralizes the impulsation of the heart, and the sound is heard without the transmission of the shock which takes place through the solid stethoscope. The diagnosis of endocarditis in the present case was free from difficulty, and, you may remember, was made a fortnight ago. The particular symptoms I shall describe more at length in a subsequent lecture.

